# Comparison of contrast in brightness mode and strain ultrasonography of glial brain tumours

**DOI:** 10.1186/1471-2342-12-11

**Published:** 2012-05-23

**Authors:** Tormod Selbekk, Reidar Brekken, Marit Indergaard, Ole Solheim, Geirmund Unsgård

**Affiliations:** 1 Department of Medical Technology, SINTEF, Olav Kyrres gate 9, Trondheim, Norway; 2 Faculty of Medicine, Norwegian University of Science and Technology, Olav Kyrres gate 9, Trondheim, Norway; 3 Department of Neurosurgery, St. Olav University Hospital, Olav Kyrres gate 9, Trondheim, Norway

**Keywords:** Ultrasound, Elastography, Elastogram, Strain, Brain, Neurosurgery, Brain tumours, Image contrast

## Abstract

**Background:**

Image contrast between normal tissue and brain tumours may sometimes appear to be low in intraoperative ultrasound. Ultrasound imaging of strain is an image modality that has been recently explored for intraoperative imaging of the brain. This study aims to investigate differences in image contrast between ultrasound brightness mode (B-mode) images and ultrasound strain magnitude images of brain tumours.

**Methods:**

Ultrasound radiofrequency (RF) data was acquired during surgery in 15 patients with glial tumours. The data were subsequently processed to provide strain magnitude images. The contrast in the B-mode images and the strain images was determined in assumed normal brain tissue and tumour tissue at selected regions of interest (ROI). Three measurements of contrast were done in the ultrasound data for each patient. The B-mode and strain contrasts measurements were compared using the paired samples t- test.

**Results:**

The statistical analysis of a total of 45 measurements shows that the contrasts in the strain magnitude images are significantly higher than in the conventional ultrasound B-mode images (P < 0.0001).

**Conclusions:**

The results indicate that ultrasound strain imaging provides better discrimination between normal brain tissue and glial tumour tissue than conventional ultrasound B-mode imaging. Ultrasound imaging of tissue strain therefore holds the potential of becoming a valuable adjunct to conventional intraoperative ultrasound imaging in brain tumour surgery.

## Background

Prior to modern neuroimaging, the neurosurgeon could detect pathological tissue by palpating the suspected areas of the brain during surgery. The tumour would be felt as a region with different elasticity compared to the surrounding normal brain, as such tumours most often have a firmer consistency than normal tissue. Even today when an operating microscope is used, the surgeon may palpate the tissue using the surgical instruments in order to find areas of the brain with differences in tissue hardness. This manual inspection of tissue hardness may aid to identify remaining tumour tissue that may be difficult to detect with direct visualisation using the operating microscope. Ultrasound imaging can also be used for the assessment of tissue hardness through imaging of *strain* in the tissue. Assuming that the stress applied to the tissue is uniform, the calculated and displayed strain values should in ideal circumstances be proportional to the modulus of elasticity (Young's modulus) of the tissues. The imaging technique is therefore often also referred to as *ultrasound elastography* and the corresponding images are often called *elastograms.*

Several research groups have investigated the use of ultrasound elastography in imaging brain tumours [1-3]. However, the clinical benefit of ultrasound strain imaging compared to conventional ultrasound imaging is still to be determined. The publications so far have shown only a few example images of brain tumours and have mainly demonstrated that it is feasible to generate elastograms of brain tumours. The measurements and display of strain require some form of displacement of the tissue, which means that internal or external forces need to act on the organ. Previous studies have demonstrated strain images (elastograms) generated by the internal displacements in the brain parenchyma caused by arterial pulsation, or generated by the use of either a mechanical shaker device or manual palpation to induce tissue displacements [1-3]. Quantitative assessments of ultrasound strain images of brain tumours have been performed in one study, which used the natural pulsation of the brain parenchyma to generate strain images. The study concluded that strain magnitudes of brain tumours are significantly lower than the strain magnitudes of normal brain tissue [4]. However, quantitative comparisons between ultrasound *strain images* and conventional *brightness mode images* have not been published so far.

The clinical performance of ultrasound strain imaging has been evaluated more thoroughly in breast tumours. In a study by Burnside *et al.,* the use of ultrasound strain imaging in combination with ultrasound B-mode imaging led to a higher area under the receiver operating characteristics (ROC) curve then by using ultrasound B- mode images alone [5].

A few ultrasound machines have implemented the option of calculating the *strain ratio* between strain levels in assumed tumour and in assumed normal tissue. This has resulted in several recent papers on the use of strain ratio (also referred to as *strain index*) for diagnostic purposes. One study compared the performance of B-mode images, strain images and strain ratio in differentiating benign and malignant tumours in a group of 227 women with focal breast lesions [6]. The authors concluded that B-mode images provided the highest sensitivity, but the strain images and strain ratio provided higher specificity. In another study Cho *et al.* compared the diagnostic performance of ultrasound B-mode sonography and strain rate in differentiation of malignant and benign breast masses and found no significant difference in the area under the ROC curve [7].

These and other studies indicate that ultrasound strain imaging may to some extent provide an improvement in diagnostics of some tumours compared to using conventional ultrasound alone. It might be asked what features of strain imaging do account for an increase in diagnostic performance compared to conventional B-mode imaging. Ultrasound is able to produce B-mode images with high spatial and temporal resolution, but the contrast resolution may be limited compared to other imaging techniques like Magnetic Resonance Imaging (MRI) or Computed Tomography (CT). The difference in brightness intensity between the lesion to diagnose and the normal tissue may in some cases appear to be low, thus having a poor contrast resolution. An improved image contrast would probably lead to improved diagnostics of these low-contrast lesions. It could be speculated that the ultrasound strain images might possess a higher image contrast than conventional ultrasound. The imaging of strain is related to other properties of tissue than the generation of ultrasound B-mode images, and therefore holds the potential to provide unique information about tissue pathology [8].

It is therefore of clinical interest to make comparisons of attributes like image contrast between the two modalities, to assess potential differences in imaging of lesions.

In this study we have processed and analysed ultrasound data acquired during surgery of glial tumours in order to compare the image contrast in conventional ultrasound images (B-mode) versus the image contrast in ultrasound strain images. We have performed a quantitative comparison of image contrast between ultrasound strain images generated by the natural pulsation of the brain parenchyma and the corresponding B-mode images. The measurements of image contrast have been performed in the peripheral parts of the tumour, covering the transition from cancerous tissue towards more normal brain tissue. The hypothesis of the study was that the contrast between tumour and normal tissue is higher in the ultrasound strain images than in the ultrasound B-mode images.

## Methods

Regional Research Ethics Committee of Central Norway approved the study protocol and the use of previously acquired ultrasound data in this retrospective study. The anonymous ultrasound data used in this study has been acquired with the patient's informed consent as a part of a prior study.

### Data acquisition

Ultrasound radiofrequency (RF) data was acquired during surgery of 15 glial tumours. The patients were diagnosed by histopathology. Eight patients had low-grade glioma (WHO-grade I and II) and 7 patients had high-grade astrocytoma (WHO grade III & IV). The data were acquired after craniotomy with the 10 MHz flat linear probe (System FiVE, GE Vingmed, Horten, Norway) kept motionless on intact dura. An engineer (TS) adjusted the settings of the ultrasound scanner (power, gain, time gain control-TGC) prior to acquisition, aiming to provide ultrasound B-mode images with a homogenous appearance but avoiding brightness saturation. The acquired data covered at least one cardiac cycle in time.

### Strain processing

The axial strain was calculated by differentiation of time delays that were estimated by processing of the ultrasound RF-data. The time delays were calculated by implementing in Matlab (MathWorks, Natic, MA, USA) a method initially suggested by Cabot [9], which has been further refined for time delay estimation in later publications [10,11]. The strain processing of the RF-data is described in details in an earlier publication from our research group [1].

### Performing the measurements

The B-mode and strain magnitude images were analysed with measuring methods implemented in Matlab. For each dataset a total of three measurements of contrast for both strain and B-mode were done at three different locations in the images. That is, a different location in the image was selected for each measurement. All three measurements for a given tumour were done at the same image frame, i.e. at the same point in time. With reference to the B-mode images, the measurements were performed in the hyperechoic tumour and the surrounding isoechoic regions presumably representing normal brain tissue.

**Figure 1:**
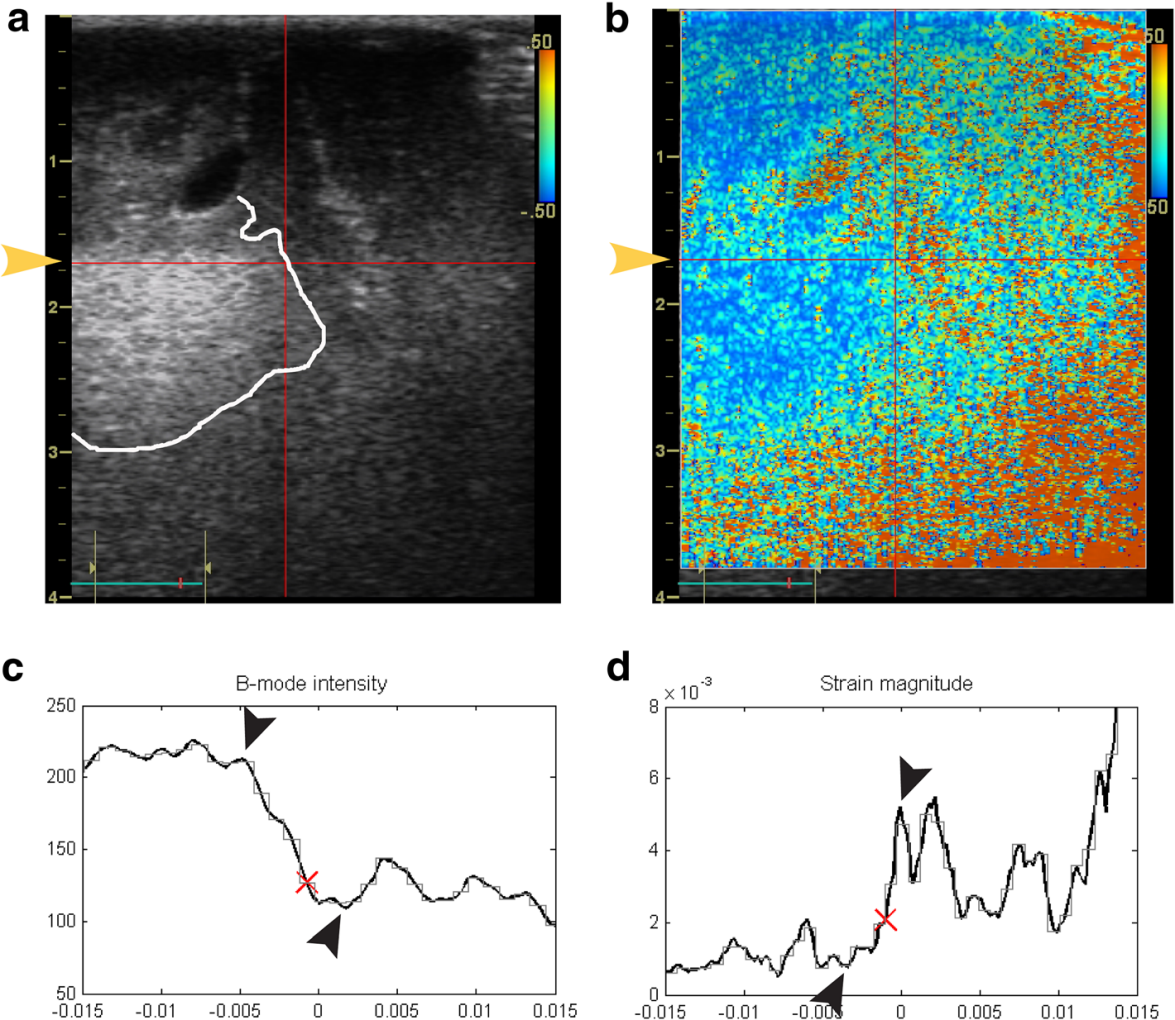
**Ultrasound B-mode and strain images.** The ultrasound B-mode image of a glioblastoma (grade IV) in **(a)** and the corresponding strain magnitude image **(b)**. For display purposes the delineation of the solid tumour close to the location of the measurements is marked with a bright line in the B-mode image. The B-mode intensity plotted along the lateral direction at a user- selected depth is shown in **(c)**, with the numbers of the x-axis referring to the distance [m] from the lateral midpoint of the image. The strain magnitudes are plotted in similar way and displayed in **(d)**, with the Y-axis showing strain magnitude values from 0–8 ‰. The depth of the plotted magnitudes is for display purposes indicated with a bright arrow in **(a)** and **(b)**. The values picked for estimation of contrast between tumour tissue and assumed normal tissue are indicated by black arrowheads in **(c)** and **(d)**.

The procedure for obtaining the contrast measurements is illustrated in Figure 1. After import and strain processing of the ultrasound data, the B-mode image was displayed. As a first step, the operator (MI) identified the tumour border as seen in the ultrasound B-mode images, and selected one position (using the mouse) on the border for contrast measurement. The B-mode intensity and strain magnitude were subsequently plotted along the lateral direction for the given depth, with a cross mark (X) indicating the lateral position of the point selected by the operator (Figure 1C-D). The plotted strain magnitudes and B-mode intensity were calculated by averaging the values over an area of approx. 1 mm^2^ in the images.

The second step of the measurements was to calculate the contrast between expected cancerous tissue and normal tissue for both image modalities. The calculation was based on the local minimum and maximum values found closest to the cross mark (X) in the respective plotted curves, i.e. the local extrema close to the tumour border as identified by the operator.

The maximum allowable lateral range for the amplitude picking was defined with the aid of a low-pass (LP) filtered version of the curves (shown as a light grey step-wise curve in Figure 1C-D), in order to increase the robustness towards minor amplitude deviations. The valid lateral range was between the first minimum and first maximum values for the LP-curve found locally around the user-selected position (marked X) of the tumour border as seen in the ultrasound images. The local extrema of the original and unfiltered curves within this lateral range were found by manual inspection, and used for the calculation of contrast. Thus, the contrast was calculated by selecting one extremum value in the tumour and a second extremum value in the supposedly normal brain tissue. The contrast between the assumed normal tissue and the tumour tissue for the two image modalities was calculated as

(1) $$C=\frac{\left|A_{1}-A_{2}\right|}{A_{1}+A_{2}}$$

where A_1_ and A_2_ are the respective local minimum and maximum values for strain magnitude or brightness intensity observed across the tumour border as depicted by ultrasound. A value of C = 0 means no difference in contrast between areas of tumour and areas of assumed normal brain tissue.

### Statistics

The differences in contrast between ultrasound strain and B-mode were statistically analysed using the paired samples t-test (SPSS Statistics, v 19.0, IBM corporation, NY, USA), using a significance level α = 0.05. Normal quantile plots (Q-Q plots) were used for assessment of the sample populations' probability distribution. The independent samples t-test was used to investigate differences in contrast between the subgroups of low-grade and high-grade gliomas for a given image modality.

## Results

**Figure 2:**
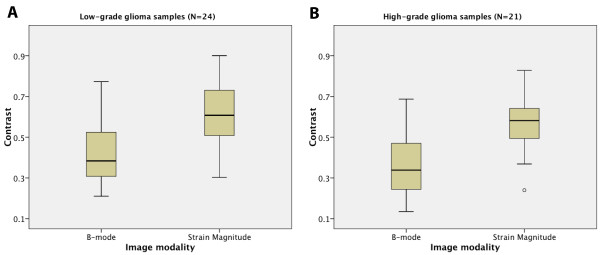
**Box plot of B-mode and strain contrast.** Box plot of brightness mode and strain magnitude contrast for the low-grade glioma samples **(a)** and the high-grade glioma samples **(b)**. The lower and upper edges of the box represent the first and third quartile respectively, while a horizontal line within the box indicates the median. The vertical length of the box represents the interquartile range (IQR). The most extreme sample values (within a distance of 1.5 IQR from the median) are the endpoints of the lines extending from the box. Possible outliers are shown as circles.

For each glial tumour (15 cases) three analyses of contrast were done on ultrasound strain magnitude and B-mode images, giving a total of 45 measurements for each modality. The measurements were performed at depths between 0.7 and 3.0 cm, with the average measurement depth being 1.9 cm for the 45 samples. Table 1 shows the average contrast and standard deviation of the three measurements performed for each patient. The average contrast is higher for the strain magnitude than for the B-mode intensity in all cases except one with a low-grade glioma. Box plot of the strain and B-mode contrast measurements is shown for the subgroups of low-grade and high-grade gliomas (Figure 2).

**Table 1:** Strain and B-mode contrast

**Glioma grading**	**Dataset No.**	**Average strain magnitude contrast (σ)**	**Average B-mode contrast (σ)**
low-grade glioma	1	0.55 (0.11)	0.43 (0.20)
	2	0.56 (0.27)	0.61 (0.20)
	3	0.57 (0.08)	0.27 (0.04)
	4	0.58 (0.19)	0.46 (0.13)
	5	0.51 (0.04)	0.32 (0.06)
	6	0.64 (0.12)	0.36 (0.07)
	7	0.66 (0.25)	0.35 (0.12)
	8	0.84 (0.04)	0.56 (0.11)
high-grade glioma	9	0.58 (0.22)	0.31 (0.06)
	10	0.48 (0.21)	0.24 (0.09)
	11	0.58 (0.11)	0.25 (0.19)
	12	0.50 (0.07)	0.29 (0.06)
	13	0.69 (0.17)	0.42 (0.08)
	14	0.65 (0.17)	0.60 (0.12)
	15	0.60 (0.03)	0.44 (0.24)
	All data	0.60 (0.16)	0.39 (0.16)

The average and standard deviation (σ) of the three contrast measurements obtained for each patient.

The difference in contrast between the two image modalities was statistically investigated for all contrast measurements of the tumours (N = 45), and for the measurements in the subgroups low-grade gliomas (N_l_ = 24) and high-grade gliomas (N_h_ = 21). The normal probability distributions of the sample populations were confirmed by inspection of Q-Q-plots. For the glial tumours as a whole the contrasts between tumour and presumably normal tissue in the strain images were significantly higher than the corresponding contrast in the B-mode images (P < 0.0001). For the subgroup of patients with high-grade gliomas the contrast in the strain images were significantly higher than the contrast in the B-mode images (P < 0.0001), and the same was observed for the subgroup with low-grade gliomas (P < 0.0001).

There was not a significant difference in contrast between the two subgroups’ low-grade and high-grade gliomas, neither for the strain images (P = 0.49) nor the B-mode images (P = 0.25).

## Discussion

In this study we have performed measurements of strain magnitude and brightness intensity across the ultrasound depicted border of glial tumours, with subsequent analysis of differences in contrast between the image modalities. The results of the analyses show a significantly higher contrast between tumour tissue and presumed normal tissue in the strain images, as compared to the B-mode images. From Table 1 we observe that the mean contrast for all B-mode measurements is 0.39 while it is 0.60 for the strain magnitude measurements, which is 54 % higher. One interpretation of the results could be that ultrasound strain imaging should be the preferred image modality to use during surgery of brain tumours, since the strain images provide better discrimination (higher contrast) between the tumour tissue and the normal brain tissue. However, in a clinical setting there are still several key issues to solve before the surgeons can use ultrasound strain imaging as a practical tool for identification of the resectable tumour tissue. With the processing parameters applied in this study the strain images generally appear noisier than the conventional B-mode images. This is partly introduced by the processing of the data where e.g. the differentiation of the calculated time delays in the axial direction typically introduces strain values with alternating polarity and a spiking appearance in the strain image. The processing is also prone to decorrelation of the echo signal due to low signal levels (hypoechoic regions in the B-mode image) or "out of plane" tissue motion causing loss of temporally coherent signals. This may cause the processing to produce false results with abnormally high strain values. Also, our method for estimation of time delays assumes that the delay is smaller than the sampling time Ts, i.e. that the tissue velocity is low compared to the number of frames acquired per second. If this assumption is not met the processing may produce incorrectly high strain values.

In our processed strain images we have indeed seen that noise can be present in parts of the image. This is typically seen in regions with low intensity in the B- mode image, for example when imaging homogenous tissue like the brain stem and deeper white brain matter that appear hypoechoic compared to other brain tissue. However, our measurements are intentionally performed in the transition zone from tumour to presumed normal brain tissue. In this short distal range we expect the data to be least influenced by noise, with the B-mode intensity ranging from the hyperechoic tumour to the isoechoic areas with presumed normal tissue. The inspection of the strain magnitude curves did not indicate any abrupt change of signal level within the spatial distance analysed, as could be expected if the strain processing produced invalid results.

It can be argued that the measurements performed in the transition zone from tumour to normal tissue impose a selection bias for the contrast analysis. This is the region that is of interest to the surgeon, but it is also the region where we should expect the strain images to be least affected by noise. The contrast measurements are only valid for analysis of image contrast between glial tumour tissue and adjacent normal tissue. It should not be interpreted to represent differences in contrast resolution between the image modalities in general.

The methodology for the analysis of image contrast in the peripheral parts of tumour involves a subjective assessment of the approximate position of the depicted tumour border and manual reading of the displayed strain magnitude and brightness curves. Even if the implemented method of analysis is not fully automatic, the measurements were obtained by following a standardized procedure, as outlined in the Methods section. Quantitative image quality measures will usually imply some subjective decisions about where to perform the analysis in the image. It is therefore difficult to establish a method without some kind of manual intervention. However, the calculation of additional measures like e.g. the contrast-to-noise ratio (CNR), or signal-to-noise ratio (SNR) would increase the robustness of the image assessment and should be considered in future studies [12]. It would also have been interesting to address intra- and interobserver variability of the measurements, which was not performed in this study.

As discussed above there are different factors that may have affected the measurements. However, we have found the obtained measurements to be quite robust and we believe that the differences in contrast found between ultrasound strain magnitude and B-mode intensity should represent actual differences between the image modalities.

Ultrasound strain imaging in brain surgery is a quite novel approach and we have not found other studies performing a similar comparison between strain images and conventional B-mode images. It is therefore difficult to compare our results with previous findings. Some studies have however explored the use of strain ratio for diagnostic purposes, but the similar ratio for B-mode intensity has not been reported. The strain ratio is a quantitative index but should not be considered as an objective *diagnostic* parameter as its value may be heavily dependent on which regions are selected for comparison and is therefore prone to variations between observers and within the patient population, which has also been pointed out by others [13]. It should be noted that the contrasts calculated in our study are not intended to serve a diagnostic purpose; the sole purpose is the pairwise comparison between the ultrasound modalities.

The *diagnostic value* of ultrasound strain imaging of brain tumours has not been assessed in this study. This would require a comparison between image findings and histology, which was not available for the current study. Glial tumours are diffuse infiltrating and tumour cells are likely to be present also beyond the border zone seen in the ultrasound B-mode image [14]. Scattered tumour cells are likely to be present in the isoechoic regions interpreted to be mainly normal brain tissue, but to a substantially less extent than in the hyperechoic regions. The calculated contrasts should therefore represent differences in magnitude (strain/brightness) between areas in the brain predominated by glial tumour cells and areas predominated by normal brain cells, respectively.

The results obtained should provide a rationale for further technical developments and investigations of methods for real-time intraoperative ultrasound strain imaging of brain tumours. The ultrasound strain magnitude images possess a higher contrast between tumour and normal brain tissue in the peripheral parts of the tumour than the conventional B-mode images. This suggest that the surgeon may use imaging of strain to improve detection of remaining tumour towards the end of surgery, compared to using conventional ultrasound imaging alone.

## Conclusions

Off-line processing of ultrasound RF-data to yield strain magnitude images has been performed on *in vivo* data acquired during brain tumour surgery. The strain magnitude images have a significantly higher contrast between normal tissue and tumour tissue than conventional B-mode images. We conclude that for glial brain tumours, ultrasound imaging of strain holds the potential to become a valuable adjunct to conventional brightness mode imaging. However, the practical aspects of acquisition and display of strain images in real time as well as evaluation of diagnostic value must be addressed in future studies.

## Competing interests

The authors declare that they have no competing interests.

## Authors' contributions

TS contributed to the study design, acquisition of data, data analyses and drafting of manuscript, RB contributed to the study design and implementation of the strain processing methods and measurements method in Matlab, MI performed the measurements and contributed to the statistical analyses and drafting of manuscript, OS contributed to data acquisition and drafting of manuscript and GU contributed to the study design, acquisition of data and drafting of manuscript. All authors read and approved the final manuscript.

## Pre-publication history

The pre-publication history for this paper can be accessed here:

http://www.biomedcentral.com/1471-2342/12/11/prepub

## References

[B1] Selbekk T Bang J Unsgaard G Strain processing of intraoperative ultrasound images of brain tumours: initial results Ultrasound Med Biol 2005 31 1 45 51 10.1016/j.ultrasmedbio.2004.09.011 15653230

[B2] Scholz M Noack V Pechlivanis I Engelhardt M Fricke B Linstedt U Brendel B Ing D Schmieder K Ermert H Harders A Vibrography during tumor neurosurgery J Ultrasound Med 2005 24 7 985 992 15972713 10.7863/jum.2005.24.7.985

[B3] Uff CE Garcia L Fromageau J Dorward N Bamber JC Real time ultrasound elastography in neurosurgery Proceedings of the IEEE International Ultrasonics Symposium 2009 467 470

[B4] Selbekk T Brekken R Solheim O Lydersen S Hernes TAN Unsgard G Tissue motion and strain in the human brain assessed by intraoperative ultrasound in glioma patients Ultrasound Med Biol 2010 36 1 2 10 10.1016/j.ultrasmedbio.2009.05.007 19854562

[B5] Burnside ES Hall TJ Sommer AM Hesley GK Sisney GA Svensson WE Fine JP Jiang J Hangiandreou NJ Differentiating benign from malignant solid breast masses with US strain imaging Radiology 2007 245 2 401 10.1148/radiol.2452061805 17940302

[B6] Thomas A Degenhardt F Farrokh A Wojcinski S Slowinski T Fischer T Significant differentiation of focal breast lesions: calculation of strain ratio in breast sonoelastography Acad Radiol 2010 17 5 558 563 10.1016/j.acra.2009.12.006 20171905

[B7] Cho N Moon WK Kim HY Chang JM Park SH Lyou CY Sonoelastographic strain index for differentiation of benign and malignant nonpalpable breast masses J Ultrasound Med 2010 29 1 1 7 20040770 10.7863/jum.2010.29.1.1

[B8] Wells PNT Liang HD Medical Ultrasound: imaging of soft tissue strain and elasticity J R Soc Interface 2011 8 64 1521 1549 10.1098/rsif.2011.0054 21680780 PMC3177611

[B9] Cabot RC A note on the application of Hilbert transform to time delay estimation IEEE Trans. Acoust. Speech Signal Processing 1981 29 607 609 ASSP 10.1109/TASSP.1981.1163564

[B10] Loupas T Powers JT Gill RW An axial velocity estimator for ultrasound blood flow imaging, based on a full evaluation of the Doppler equation by means of a two-dimensional autocorrelation approach IEEE Trans Ultrason Ferroelect Freq Control 1995 42 672 688

[B11] Simon C VanBaren P Ebbini ES Two-dimensional temperature estimation using diagnostic ultrasound IEEE Trans Ultrason Ferroelect Freq Control 1998 45 1088 1099 10.1109/58.710592 18244264

[B12] Varghese T Ophir J An analysis of elastographic contrast-to-noise ratio Ultrasound Med Biol 1998 24 915 924 10.1016/S0301-5629(98)00047-7 9740393

[B13] Kagoya R Monobe H Tojima H Utility of elastography for differential diagnosis of benign and malignant thyroid nodules Otolaryngol Head Neck Surg 2010 143 2 230 234 10.1016/j.otohns.2010.04.006 20647125

[B14] Unsgaard G Selbekk T Müller TB Ommedal S Torp SH Myhr G Bang J Hernes TAN Ability of navigated 3D ultrasound to delineate gliomas and metastases - comparison of image interpretations with histopathology Acta Neurochir 2005 147 12 1259 1269 10.1007/s00701-005-0624-1 16172831

